# Aripiprazole as a risk factor for impulse control disorders: a systematic review

**DOI:** 10.1192/bjo.2021.795

**Published:** 2021-06-18

**Authors:** Benjamin Williams, Benjamin Williams, Kishen Neelam, Saumya Singh

**Affiliations:** 1 Mental Health Liaison Team, Blackpool Victoria Hospital; 2 Barnett House, Greater Manchester Mental Health NHS FT; 3 Bolton North CMHT, Bentley House, Greater Manchester Mental Health NHS FT

## Abstract

**Aims:**

Aripiprazole is an anti-psychotic medication widely used for bipolar affective disorder and depression. It's primary mechanism of action is as a partial dopamine agonist. Aripiprazole's effect on dopamine signalling in the mesolimbic and mesocortical pathways may lead to impulse control disorders, as seen with other dopamine agonist medications. Aripiprazole is often chosen by prescribers because of its favourable side effect profile. There is a need to synthesise the available epidemiological literature on the potential association between aripiprazole use and impulse control disorders. This is needed to inform patients and prescribers of the best available evidence regarding this potential association. Our aim is to conduct a systematic review of the available non case-study evidence on the potential association between aripiprazole and impulse control disorders.

**Method:**

Databases were searched using MEDLINE, PsychINFO, EMBASE, Cochrane Clinical Trials and Web of science. All studies from no earliest date to December 2020 were included; adult patients with a severe and enduring mental illness prescribed antipsychotic medication were included. Cinician diagnosis, structured interview diagnosis, and interviewer or self-completion questionnaires were used to measure prevalence. The study designs included were experimental designs, cohort study, cross-sectional survey and administrative databases. Exclusion criteria being those with traumatic brain injury, psychosis secondary to autoimmune, iatrogenic, chromosomal or metabolic disorder, those with Learning disability or Autistic Spectrum disorders. studies with majority of participants <18yrs. Those who were on other antipsychotic medications in addition to Aripiprazole, were excluded. To ensure quality assurance, we used ROBINS-I tool and GRADE assessment to measure the risk of bias.

**Result:**

240 records were retrieved, 187 after duplicates were removed. 8 full text articles were assessed for eligibility, of which 4 were included in the qualitative synthesis. 2 studies were analyses of spontaneous adverse drug reaction reporting systems and 2 of health insurance claims databases. All 4 studies found aripiprazole to be associated with greater risk of impulse control disorders. The single study which compared directly with other antipsychotics had a much smaller effect size. Study heterogeneity precluded meta-analysis. All studies were at high risk of bias. The quality of evidence is very low.

**Conclusion:**

The available evidence is consistent with the existing warnings regarding increased risk of impulse control disorders in patients prescribed aripiprazole. Clinicians may wish to monitor for this adverse drug reaction. Further research which can account for potential confounders, examines specific impulse control disorders and which is less susceptible to detection and ascertainment biases is required.

